# Case Report: Acral *MYH10::ALK* fusion superficial mesenchymal neoplasm: first proof of metastatic potential

**DOI:** 10.3389/fonc.2026.1835779

**Published:** 2026-06-02

**Authors:** Masih Kameh Khosh, Sascha Wellenbrock, Daniel Kameh Khosh, Tobias Hirsch, Abbas Agaimy, Dan Mon O'Dey

**Affiliations:** 1 Department of Plastic and Reconstructive Surgery, University Hospital Muenster, Muenster, Germany; 2 Department of Plastic, Reconstructive and Aesthetic Surgery, Hand Surgery, Hornheide Specialist Clinic, Muenster, Germany; 3 Faculty of Medicine and Health Sciences, University of Antwerp, Antwerp, Belgium; 4 Institute of Pathology, Friedrich-Alexander-University Erlangen-Nuernberg, University Hospital, Erlangen, Germany; 5 O'Dey Private Practice: Plastic & Hand Surgery, Salutem Private Clinic, Heidelberg, Germany

**Keywords:** acral tumors, ALK fusion, mesenchymal tumor, metastatic potential, rare entity

## Abstract

**Background:**

Superficial *ALK-*rearranged soft tissue tumors are rare mesenchymal neoplasms showing heterogenous morphology and clinical behavior. They usually present as slowly enlarging nodules in young adults and can mimic other spindle cell tumors. While some *ALK*-altered mesenchymal neoplasms can be classified into defined WHO categories (e.g., inflammatory myofibroblastic tumor, epithelioid fibrous histiocytoma, and a few others), the nosology of many tumors has not yet been delineated, and they are currently reported descriptively. A case of a young man with a three-year follow-up is presented, providing prognostic insights into this entity.

**Case description:**

A healthy 23-year-old male presented with a purplish mass on the plantar left foot that had enlarged over one year. MRI showed a well-defined lesion without bone involvement. Histopathology revealed sheets of bland round to oval cells within myxohyaline stroma containing focal pseudorosette-like hyalinized nodules and increased mitotic activity. Immunohistochemistry demonstrated strong cytoplasmic ALK expression with heterogeneous expression for CD34, and S100. Focal EMA and pancytokeratin expression were noted. Next-generation sequencing confirmed a *MYH10::ALK* fusion. After excision with positive margins, re-excision revealed residual tumor as well. Further surgery was declined, and the patient developed metastatic disease during follow-up.

**Discussion:**

This case broadens the spectrum of acral *ALK*-rearranged unclassified mesenchymal tumors and identifies *MYH10* as a potential fusion partner in metastatic disease. Although these tumors are typically indolent, features such as increased mitotic activity may indicate more aggressive behavior. Diagnosis requires integrated histologic, immunophenotypic, and molecular assessment. This case highlights the metastatic potential of *ALK*-rearranged unclassified mesenchymal neoplasms, particularly those exhibiting a tyrosine kinase phenotype (co-expression of S100 and CD34), supporting their classification within the intermediate, rarely metastasizing category according to soft tissue tumor stratification principles. It further emphasizes the need for heightened clinical vigilance and thorough surgical management, which may influence long-term outcomes.

## 1 Introduction

Soft tissue tumors with *ALK* gene rearrangements represent a recently recognized and heterogeneous group of mesenchymal neoplasms, with emerging evidence delineating their clinicopathologic and molecular spectrum (1–3). These tumors may present in young adults as slowly enlarging, often purplish nodules or masses, and can be challenging to diagnose due to their rarity and overlapping features with other spindle cell neoplasms (4, 5).

Histopathologically, *ALK*-rearranged tumors may either fit within a defined WHO category (e.g., inflammatory myofibroblastic tumor, epithelioid fibrous histiocytoma) or represent an unclassified, highly heterogeneous group of cutaneous and subcutaneous tumors. These tumors frequently display a myxoid or myxohyaline stroma with spindle to ovoid cell morphology, or epithelioid features with or without high-grade cytology. These tumors have been referred to by variable and often vaguely defined terms: some have been grouped with epithelioid fibrous histiocytomas, others described as superficial *ALK*-altered myxoid spindle cell neoplasms (SAMS), and many additional cases have been reported descriptively as exhibiting epithelioid or myxoid morphology. Immunophenotypically, they are characterized by predominantly cytoplasmic *ALK* expression without additional lineage-specific marker expression. However, a subset of tumors shows co-expression of CD34 and S100 protein, analogous to other tyrosine kinase fusion–driven neoplasms, such as those harboring *NTRK* fusions (1, 3). The biological behavior of these tumors remains incompletely defined. Limited follow-up data suggests a generally indolent course; however, occasional recurrences and metastases have been reported, underscoring the need for careful clinicopathologic correlation and long-term surveillance. This case contributes to the growing body of literature on *ALK*-fused mesenchymal neoplasms, particularly those arising in acral locations, with several years of follow-up offering valuable insight into long-term behavior and clinical outcomes. This report has been prepared in accordance with the CARE guidelines (6).

## 2 Case description

A healthy 23-year-old male with no relevant medical history presented with a 2.5 x 2.5 cm tumor on the left foot. The sharply demarcated, purplish mass had a firm, rubbery consistency and was located on the plantar aspect of the first metatarsophalangeal joint (**Figure 1**). The patient first noticed the mass approximately one year ago, and it had progressively enlarged, causing increasing discomfort while walking. The delay in seeking medical attention was attributed to the patient’s apprehension about visiting healthcare professionals. MRI revealed no immediate contact between the mass and the underlying bone (**Figure 2**). Surgical resection was performed, followed by histopathological examination (**Figures 3**, **4**).

**Figure 1:**
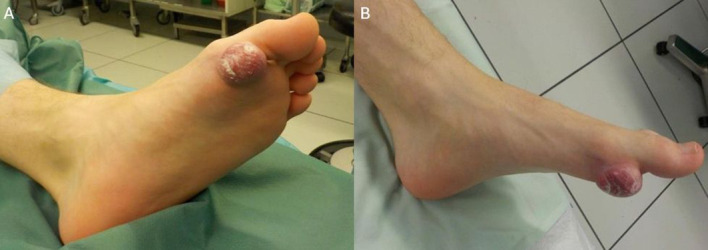
Clinical imaging demonstrating a smooth, purplish tumor near the base of the left first toe. **(A)** Plantar view. **(B)** Side view.

**Figure 2:**
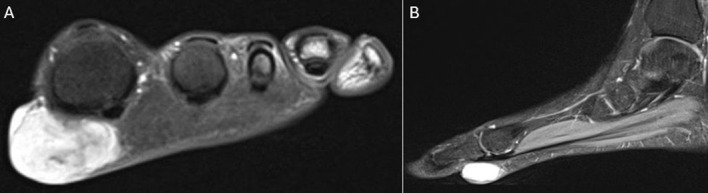
MRI demonstrating a soft-tissue tumor at the level of the first metatarsophalangeal joint without bone involvement. **(A)** Coronal view. **(B)** Sagittal view.

**Figure 3:**
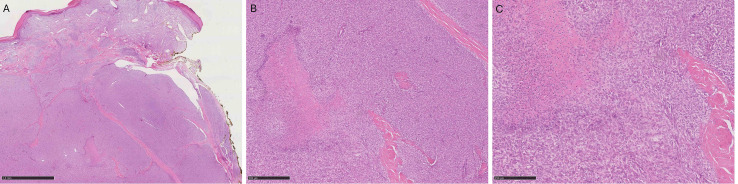
Tumor microscopy: hematoxylin-eosin (H&E) staining. **(A)** 12,5× magnification. **(B)** 50× magnification. **(C)** 100× magnification.

**Figure 4:**
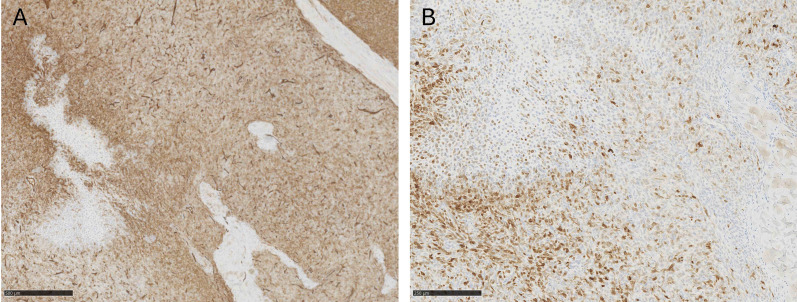
Tumor microscopy: Immunohistochemical staining for CD34 and S100. **(A)** CD34 immunohistochemical staining (50× magnification). **(B)** S100 immunohistochemical staining (100× magnification).

### 2.1 Timeline of clinical events

## 3 Diagnostics and therapy

Microscopic examination demonstrated tumor involvement of the dermis, subcutaneous tissue, and fascia, including focal invasion into adjacent skeletal muscle. Microscopic involvement of the surgical margins was noted. The lesion exhibited densely cellular proliferation of round to oval cells with large vesicular nuclei and poorly delineated cytoplasm. Repeated cell condensation around small blood vessels was identified, with no evidence of multinucleated cell forms. Mitotic activity was substantially increased, with up to 7 mitoses per 2 mm (2) and small areas of necrosis were present. Additionally, sclerotic stromal areas containing smaller epithelioid cells were observed. Molecular analysis was performed using, among others, next generation sequencing, identifying an in-frame *MYH10::ALK* fusion gene. Subsequently an immunohistochemical examination was carried out, which demonstrated a strong cytoplasmic positivity for ALK (clone 1A4), in line with the detected *ALK* gene fusion. Furthermore, the tumor cells were positive for S100 and CD34 with focal expression of pancytokeratin and with detectable vessel invasion.

The biological behavior and prognostic implications of the tumor were uncertain, and after interdisciplinary discussion, a recommendation for a repeat surgical resection was made, to which the patient consented following multiple consultations. The procedure took place three months after the initial operation, uncovering a small, residual tumor adhering to the dermis during intraoperative examination. Histopathology once again identified microscopic residual tumor medially, laterally and dorsally. Despite thorough consultation, the patient decided against further resection, opting instead for a wait-and-see approach under the supervision of an oncologist with periodic MRI exams of the foot. Following an initially unremarkable clinical and radiological course with no evidence of macroscopic tumor recurrence, the patient presented twelve months later with new-onset left inguinal lymphadenopathy. Imaging revealed suspicious lymph nodes in the groin, as well as lesions of the hip involving the piriformis and iliopsoas muscles. Staging further demonstrated likely malignant pulmonary lesions in the left upper lobe and the right lower lobe. Subsequent inguinal lymphadenectomy confirmed lymph node metastasis of the tumor (pT1 pN1 cM1). The patient declined surgical removal of the other lesions, including those in the hip musculature, and systemic therapy with crizotinib 500 mg daily was initiated, followed by scheduled follow-up examinations. After an initial phase of stable disease, imaging at 10 months revealed progression of the hip musculature lesions, prompting treatment with locoregional radiotherapy. Recent imaging demonstrates a substantial reduction in the size of these lesions, with no further signs of disease progression. **Table 1** shows the timeline of clinical events.

**Table 1:** Timeline of clinical events.

Date	Clinical event
Jan 2023	Initial presentation with a progressively enlarging left foot tumor over one year.
Apr 2023	Surgical tumor excision with positive margins.
Aug 2023	Re-excision
Jul 2024	Presentation with left inguinal lymphadenopathy, further imaging initiated
Aug 2024	Lymphadenectomy performed, lymph node metastasis confirmed
Sep 2024	Surgery declined, Crizotinib therapy started
Feb 2025	Imaging shows stable disease
Jun 2025	Discovery of lesions within the left hip musculature
Jul 2025	Initiation of locoregional radiotherapy of the hip
Jan 2026	Staging shows regression of the hip lesions and otherwise stable disease.

## 4 Discussion

The present case of a 23-year-old male with an acral soft tissue tumor harboring a *MYH10::ALK* fusion exemplifies the evolving clinicopathologic spectrum and the biological potential of *ALK*-rearranged mesenchymal neoplasms. These tumors are increasingly recognized in superficial and acral locations, typically presenting in young to middle-aged adults as slowly enlarging, well-demarcated masses with variable symptoms. They may be misdiagnosed due to their rarity and overlapping features with other entities, such as spindle cell neoplasms (1–3, 7, 8). Approximately 71% of superficial tumors arise in the lower extremities, followed by the upper extremities (14%), with less frequent involvement of the trunk and head and neck regions (2). **Table 2** presents a range of differential diagnoses based on clinical and histopathological features that warrant consideration and exclusion.

**Table 2:** Differential diagnoses for acral *MYH10::ALK* fusion superficial mesenchymal neoplasm.

Differential diagnosis	Acral/plantar site predilection	Histological overlap	Key distinguishing features
Epithelioid fibrous histiocytoma (EFH) / SAMS	Predominantly lower extremity (~71%); uncommon at acral sites	ALK+, CD34-/+, S100-/+; epithelioid or spindle cell morphology, sparse fibrous or myxoid stroma	Typically dermal, cytologically bland, low mitotic activity, no necrosis; benign behavior; lacks high-grade features
Inflammatory myofibroblastic tumor (IMT)	Rare in skin, even rarer at acral sites	ALK+ (~75%); spindle cells in fibromyxoid stroma with inflammatory infiltrate	Desmin-/+, SMA+; prominent lymphoplasmacytic infiltrate; CD34−, S100−; characteristic fusion partners (e.g., TPM3)
NTRK-rearranged spindle cell neoplasm	Occurs in extremities, including acral sites	Co-expression of S100 and CD34; perivascular hyalinization	SOX10− NTRK1/2/3; ALK− by IHC; requires molecular confirmation
Clear cell sarcoma	Predilection for tendons/aponeuroses of foot and ankle in young adults	S100+; epithelioid & spindle cells in nests within fibrous septa.	HMB-45+, Melan-A+, SOX10+; EWSR1::ATF1 or EWSR1::CREB1/CREM fusion; CD34−; melanocytic differentiation
Synovial sarcoma	Common malignant soft tissue tumor of the foot	Spindle and/or round cells; rarely biphasic	EMA+, cytokeratin+/-; SS18::SSX fusion; CD34−; biphasic or monophasic architecture; SS18 IHC+; SS18::SSX1/2 fusions
Superficial CD34-positive fibroblastic tumor	Predominantly lower extremity (~73%)	CD34+; spindle pleomorphic cells in superficial soft tissue	Glassy cytoplasm, nuclear inclusions; PRDM10 rearrangement; S100−; CADM3+, WT1+; ALK−
Extraskeletal myxoid chondrosarcoma	Rare in acral/plantar sites; case reports in plantar fascia	Myxoid stroma with cords/nests of round-to-spindled cells; variable S100	S100+, CD117+/-, synaptophysin+/-, INSM1+, p63+/-; NR4A3 rearrangement (e.g., EWSR1::NR4A3; rarely TAF15 & others); CD34−; lobulated architecture; indolent but metastatic potential

Histologically, *ALK*-rearranged tumors exhibit a broad morphologic spectrum, ranging from well-defined entities (e.g., inflammatory myofibroblastic tumor, epithelioid fibrous histiocytoma) to unclassified, heterogeneous neoplasms characterized by spindle to epithelioid cell patterns, myxoid or myxohyaline stroma, and variable cytologic features, spanning from predominantly bland-appearing to, in rare cases, frankly malignant cytology. The latter may show increased mitotic activity and focal necrosis, as seen in this case. Pediatric cases of fibrosarcoma-like spindle cell tumors of the soft tissue and kidney harboring *ALK* fusions further support the concept that *ALK*-rearranged spindle cell neoplasms constitute a broader, molecularly defined entity with overlapping clinicopathologic features across age groups and anatomic sites (9). Immunophenotypically, co-expression of ALK, CD34, and S100, as seen in this case, is characteristic of a subgroup of unclassified *ALK*-rearranged tumors, that are part of the emerging family of tyrosine kinase fusion-positive mesenchymal neoplasms. They occur across all age groups (infancy to elderly) and can involve superficial and deep soft tissue as well as viscera (1, 8). *MYH10* is a recurrent partner in *ALK*-rearranged tumors and preserves the tyrosine kinase domain, which is relevant for both diagnosis and potential targeted therapy (2, 8, 10). The identification of a *MYH10*::*ALK* fusion by next-generation sequencing is a notable finding in this case. To date, only three tumors harboring this fusion have been reported across two prior series; none occurred at an acral site, and follow-up was limited to less than 10 months in two cases without reported adverse events. No recurrences or metastases have been documented specifically for superficial tumors with *MYH10*::*ALK* fusion in the available literature (1, 8).

Molecular characterization is important for precise diagnosis and prognostication in *ALK*-associated neoplasms, particularly in recurrent or refractory cases (10–12). ALK immunohistochemistry alone cannot reliably distinguish true *ALK* fusion-driven tumors from morphologic or immunophenotypic mimics. Overly sensitive *ALK* immunohistochemistry protocols may yield false-positive or equivocal results due to background staining in non-*ALK*-rearranged tumors, and aberrant ALK expression has been increasingly recognized in neoplasms carrying non-*ALK* gene fusions, necessitating confirmatory molecular testing to accurately identify *ALK* gene rearrangements (13). Next-generation sequencing enables precise identification of fusion partners, which may have therapeutic implications (11). In this context, ALK inhibitors, such as crizotinib and alectinib, have demonstrated efficacy in select cases of *ALK*-rearranged mesenchymal tumors, particularly in advanced or metastatic disease (14).

Prognostically, most superficial *ALK*-rearranged tumors appear to follow an indolent course, with low rates of recurrence and metastasis, particularly in cases lacking high-grade features or deep tissue involvement. However, cases with increased mitotic activity, necrosis, or deep extension may exhibit more aggressive behavior, underscoring the need for careful histopathologic assessment and long-term follow-up. The presence of vascular invasion in this case was a further risk factor, as documented in select reports (1, 3, 7).

In summary, this case highlights the importance of integrating clinicopathologic, immunohistochemical, and molecular data in the diagnosis and management of acral *ALK*-rearranged soft tissue tumors. Ongoing surveillance and interdisciplinary discussion are warranted, given the potential for aggressive behavior, as illustrated in this case.

## 5 Patient perspective

### 5.1 Translated from German

“A few years ago, I noticed a slowly growing lump under my foot, which I initially tried to ignore. I was afraid of seeing a doctor and kept hoping it would disappear on its own. Even though I felt that something was not right, I continued with my daily life until I could no longer fit properly into my shoe and every step became painful.

After the tumor was removed, I hoped for quick reassurance, but instead I was faced with a lot of uncertainty. I was told that the findings were unusual and that specialists needed more time to understand what this meant. Waiting for answers was not easy, as I did not know whether the tumor was harmless or something that could become dangerous. This made it difficult to feel at ease, even though I felt physically well and the wound was healing without problems.

Following a second operation, I was told that further tissue might still need to be removed. This was overwhelming. The idea of potentially facing who-knows-how-many surgeries was frightening, especially since I was recovering well and was hoping to get back to work. I decided for close monitoring instead and tried to focus on returning to a normal routine. For some time, things were indeed stable, and I was working full-time again.

Later, I noticed new symptoms, and suddenly the situation felt more serious. The possibility that the disease had spread terrified me, but making decisions about further treatment still was not easy, particularly when surgery was discussed again, which I did not want to pursue. I am currently taking medication and have undergone radiotherapy for the spots in my hip, which have since become much smaller. Although uncertainty remains, I do not want this condition to control my life. At present, the situation feels manageable, and I am confident that if new challenges arise in the future, we will be able to deal with them as well.

## Data Availability

The original contributions presented in the study are included in the article/supplementary material. Further inquiries can be directed to the corresponding author.

## References

[1] Dermawan JK Azzato EM Goldblum JR Rubin BP Billings SD Ko JS . Superficial ALK-rearranged myxoid spindle cell neoplasm: a cutaneous soft tissue tumor with distinctive morphology and immunophenotypic profile. Mod Pathol. (2021) 34:1710–8. doi: 10.1038/s41379-021-00830-w. PMID: 34088997

[2] Dermawan JK DiNapoli SE Mullaney KA Sukhadia P Agaram NP Dickson BC . ALK-rearranged mesenchymal neoplasms: a report of 9 cases further expanding the clinicopathologic spectrum of emerging kinase fusion positive group of tumors. Genes Chromosomes Cancer. (2023) 62:75–84. doi: 10.1002/gcc.23097. PMID: 36125853 PMC10483220

[3] Ouyang Q Guo X Mao R Cao Z . Case report: ALK-rearranged mesenchymal neoplasms with S100 and CD34 co-expression: additional cases with distinct characteristics. Front Oncol. (2025) 15:1516491. doi: 10.3389/fonc.2025.1516491. PMID: 40510160 PMC12159070

[4] Angelini A Biz C Cerchiaro M Longhi V Ruggieri P . Malignant bone and soft tissue lesions of the foot. J Clin Med. (2023) 12. doi: 10.3390/jcm12083038. PMID: 37109375 PMC10146676

[5] Murahashi Y Iba K Teramoto A Emori M Okada Y Kamiya T . Clinical features of bone and soft tissue tumors of the foot and ankle: results from a retrospective single-center case-series. J Orthop Sci. (2021) 26:885–90. doi: 10.1016/j.jos.2020.08.016. PMID: 33067084

[6] Riley DS Barber MS Kienle GS Aronson JK von Schoen-Angerer T Tugwell P . CARE guidelines for case reports: explanation and elaboration document. J Clin Epidemiol. (2017) 89:218–35. doi: 10.1016/j.jclinepi.2017.04.026. PMID: 28529185

[7] Agaimy A . The expanding family of ALK-rearranged mesenchymal neoplasms. Semin Diagn Pathol. (2025) 42:150947. doi: 10.1016/j.semdp.2025.150947. PMID: 40803981

[8] Gestrich CK Davis JL Biederman L John I Alaggio R Giovannoni I . ALK-rearranged epithelioid mesenchymal neoplasm: expanding the spectrum of tyrosine kinase-altered mesenchymal tumors. Mod Pathol. (2023) 36:100334. doi: 10.1016/j.modpat.2023.100334. PMID: 37726067

[9] Tan SY Al-Ibraheemi A Ahrens WA Oesterheld JE Fanburg-Smith JC Liu YJ . ALK rearrangements in infantile fibrosarcoma-like spindle cell tumours of soft tissue and kidney. Histopathology. (2022) 80:698–707. doi: 10.1111/his.14603. PMID: 34843129

[10] Coppock JD Schneider MA Surrey LF Karakousis GC Maki RG Cooper K . S100 and CD34 expressing mesenchymal neoplasm with rare PLEKHH2::ALK fusion and response to ALK inhibition. Am J Surg Pathol. (2022) 46:1309–13. doi: 10.1097/PAS.0000000000001887. PMID: 35288525

[11] Jung M Moon KC Bae J Kim TM Kim M Jeon YK . ALK translocation in ALK-positive mesenchymal tumors: diagnostic and therapeutic insights. Arch Pathol Lab Med. (2022) 146:1460–70. doi: 10.5858/arpa.2021-0330-OA. PMID: 35438749

[12] Sadhu S Barwad A Mridha AR Malik PS Nambirajan A Jain D . ALK rearranged Malignant mesenchymal neoplasms of thorax: therapeutically targetable “ALKomas” beyond the spectrum of non-small cell lung carcinomas and thoracic inflammatory myofibroblastic tumors. Virchows Arch. (2025). doi: 10.1007/s00428-025-04198-z. PMID: 40760193

[13] Kassem I Van Essche C Hoton D Dubois D Camby P Dekairelle AF . Orthogonal validation of anaplastic lymphoma kinase (ALK) immunohistochemistry with molecular analysis for ALK gene rearrangement is required to finetune staining protocols with the D5F3 clone and can impact external quality assessment. Virchows Arch. (2025). doi: 10.1007/s00428-025-04345-6. PMID: 41354989

[14] Wu J Hu Y Abdihamid O Huang G Xiao S Li B . Crizotinib in sarcomatous Malignancies harboring ALK fusion with a definitive partner(s): response and efficacy. Front Oncol. (2021) 11:684865. doi: 10.3389/fonc.2021.684865. PMID: 34722239 PMC8551604

